# Changes to virus taxonomy and the ICTV Statutes ratified by the International Committee on Taxonomy of Viruses (2024)

**DOI:** 10.1007/s00705-024-06143-y

**Published:** 2024-11-03

**Authors:** Peter Simmonds, Evelien M. Adriaenssens, Elliot J. Lefkowitz, Hanna M. Oksanen, Stuart G. Siddell, Francisco Murilo Zerbini, Poliane Alfenas-Zerbini, Frank O. Aylward, Donald M. Dempsey, Bas E. Dutilh, Juliana Freitas-Astúa, María Laura García, R. Curtis Hendrickson, Holly R. Hughes, Sandra Junglen, Mart Krupovic, Jens H. Kuhn, Amy J. Lambert, Małgorzata Łobocka, Arcady R. Mushegian, Judit Penzes, Alejandro Reyes Muñoz, David L. Robertson, Simon Roux, Luisa Rubino, Sead Sabanadzovic, Donald B. Smith, Nobuhiro Suzuki, Dann Turner, Koenraad Van Doorslaer, Anne-Mieke Vandamme, Arvind Varsani

**Affiliations:** 1 Nuffield Department of Medicine, University of Oxford, Peter Medawar Building, South Parks Road, Oxford, OX1 3SY UK; 2 Quadram Institute Bioscience, Norwich Research Park, Norwich, NR4 7UQ UK; 3 Department of Microbiology, University of Alabama at Birmingham, BBRB 276, 845 19th St South, Birmingham, AL 35294-2170 USA; 4 Molecular and Integrative Biosciences Research Programme, Faculty of Biological and Environmental Sciences, University of Helsinki, Viikinkaari 9, 00014 Helsinki, Finland; 5 School of Cellular and Molecular Medicine, Faculty of Life Sciences, University of Bristol, University Walk, Bristol, BS8 1TD UK; 6 Departamento de Fitopatologia/BIOAGRO, Universidade Federal de Viçosa, Viçosa, MG 36570-900 Brazil; 7 Departamento de Microbiologia, Universidade Federal de Viçosa, Viçosa, MG 36570-900 Brazil; 8 Department of Biological Sciences, Virginia Tech, Blacksburg, VA USA; 9 Institute of Biodiversity, Faculty of Biological Sciences, Cluster of Excellence Balance of the Microverse, Friedrich Schiller University, Fürstengraben 1, 07743 Jena, Germany; 10 Theoretical Biology and Bioinformatics, Department of Biology, Utrecht University, Padualaan 8, 3584 CH Utrecht, The Netherlands; 11 Embrapa Cassava and Fruits, Brazilian Agricultural Research Corporation, Cruz das Almas, BA 44380-000 Brazil; 12 Instituto de Biotecnología y Biología Molecular, CCT-La Plata, CONICET-UNLP, Calles 47 y 115 (1900), La Plata, Buenos Aires, Argentina; 13 Centers for Disease Control and Prevention, Fort Collins, Colorado USA; 14 Institute of Virology, Charité-Universitätsmedizin, Corporate Member of Free University Berlin, Humboldt-University Berlin, and Berlin Institute of Health, Berlin, Germany; 15 Archaeal Virology Unit, Institut Pasteur, Université Paris Cité, CNRS UMR6047, 25 rue du Dr Roux, 75015 Paris, France; 16 Integrated Research Facility at Fort Detrick, Division of Clinical Research, National Institute of Allergy and Infectious Diseases, National Institutes of Health, B-8200 Research Plaza, Fort Detrick, Frederick, MD 21702 USA; 17 Division of Vector-Borne Diseases, Centers for Disease Control and Prevention, National Center for Emerging and Zoonotic Infectious Diseases, Fort Collins, CO 80521 USA; 18 Institute of Biochemistry and Biophysics of the Polish Academy of Sciences, 02-106 Warsaw, Poland; 19 Division of Molecular and Cellular Biosciences, National Science Foundation, 2415 Eisenhower Avenue, Alexandria, VA 22314 USA; 20 Institute for Quantitative Biomedicine, Rutgers University, Piscataway, New Jersey USA; 21 Departamento de Ciencias Biológicas, Universidad de los Andes, Bogotá, Colombia; 22 MRC-University of Glasgow Centre for Virus Research, Sir Michael Stoker Building, 464 Bearsden Road, Glasgow, G61 1QH UK; 23 DOE Joint Genome Institute, Lawrence Berkeley National Laboratory, Berkeley, California USA; 24 Consiglio Nazionale delle Ricerche, Istituto per la Protezione Sostenibile delle Piante, Sede Secondaria di Bari, Via Amendola 165/A, 70126 Bari, Italy; 25 Department of Agricultural Science and Plant Protection, Mississippi State University, Mississippi State, MS 39762 USA; 26 Institute for Genomics, Biocomputing and Biotechnology, Mississippi State University, Mississippi State, MS 39762 USA; 27 Institute of Plant Science and Resources, Okayama University, Kurashiki, Okayama 710-0046 Japan; 28 School of Applied Sciences, Faculty of Health, Science and Society, University of the West of England, Bristol, UK; 29 Department of Immunobiology, School of Animal and Comparative Biomedical Sciences, BIO5 Institute, University of Arizona Cancer Center, Tucson, AZ 85721 USA; 30 Department of Microbiology, Immunology and Transplantation, Clinical and Epidemiological Virology, Rega Institute for Medical Research, KU Leuven, 3000 Leuven, Belgium; 31 Center for Global Health and Tropical Medicine, Instituto de Higiene e Medicina Tropical, Universidade Nova de Lisboa, Rua da Junqueira, 100, 1349-008 Lisbon, Portugal; 32 The Biodesign Center for Fundamental and Applied Microbiomics, School of Life Sciences, Center for Evolution and Medicine, Arizona State University, Tempe, AZ 85287-4701 USA

## Abstract

This article reports changes to virus taxonomy and taxon nomenclature that were approved and ratified by the International Committee on Taxonomy of Viruses (ICTV) in April 2024. The entire ICTV membership was invited to vote on 203 taxonomic proposals that had been approved by the ICTV Executive Committee (EC) in July 2023 at the 55th EC meeting in Jena, Germany, or in the second EC vote in November 2023. All proposals were ratified by online vote. Taxonomic additions include one new phylum (*Ambiviricota*), one new class, nine new orders, three new suborders, 51 new families, 18 new subfamilies, 820 new genera, and 3547 new species (excluding taxa that have been abolished). Proposals to complete the process of species name replacement to the binomial (genus + species epithet) format were ratified. Currently, a total of 14,690 virus species have been established.

## Introduction

The International Committee on Taxonomy of Viruses (ICTV) follows an annual cycle of taxonomy updating in which proposed changes and additions to virus taxonomy are considered and implemented. The ICTV classification of viruses provides a framework for the taxonomic placement of viruses at ranks from species to realm and furthermore regulates their taxon names and typography. The ICTV Statutes (https://ictv.global/about/statutes) describe the process in which taxonomic proposals are submitted to the ICTV Executive Committee (EC) and undergo review with input from the ICTV Study Groups and Subcommittees, other interested virologists, and the EC. After final approval by the EC, proposals are placed on the ICTV website (https://ictv.global) for evaluation by the full ICTV membership, which ratifies them by online voting.

## Proposal discussion and ratification

The EC of the ICTV held a hybrid meeting in Jena, Germany, on the 2nd - 4th August, 2023. The EC reviewed a total of 202 taxonomy proposals submitted to the seven subcommittees, including streamlined proposals that had been reviewed previously by at least two EC members, and two general proposals. Requested revisions to 13 proposals were reviewed and re-voted on by the EC in November 2023. The 2023 taxonomy proposals that were accepted by the EC were then placed on the ICTV website (https://ictv.global) for viewing by the full ICTV membership.

All proposals [1–203] were ratified in a vote held from the 28^th^ March to the 25^th^ April, 2024. A total of 125 out of 180 ICTV members (69%) voted on the proposals. All proposals received either 120 or 121 votes (*i.e.*, 99.2% or 100%) in favour of ratification. A summary of the taxonomy changes enacted by the proposals is provided in Table 1. Each proposal is cited and listed in the References to acknowledge the authors’ efforts and to provide links to the specific proposal on the ICTV website. These documents and those from previous years are permanently available to provide full access to the text and listing of taxonomy changes made in each proposal (https://ictv.global/files/proposals/approved).

**Table 1:** Summary of ratified taxonomic changes in 2024

Rank	MSL.38 total^a^	New	Abolished	Moved	Renamed	MSL.39 total^b^	Net change
Realm	6	0	0	0	0	6	0
Subrealm	0	0	0	0	0	0	0
Kingdom	10	0	0	0	0	10	0
Subkingdom	0	0	0	0	0	0	0
Phylum	17	1	0	0	0	18	+1
Subphylum	2	0	0	0	0	2	0
Class	40	1	0	0	1	41	+1
Subclass	0	0	0	0	0	0	0
Order	72	9	0	0	3	81	+9
Suborder	8	3	0	0	0	11	+3
Family	264	51	−1	12	2	314	+50
Subfamily	182	18	0	1	1	200	+18
Genus	2818	820	−116	58	1	3522	+704
Subgenus	84	0	0	0	0	84	0
Species	11273	3547	−130	407	2884	14690	+3417

^a^Total number of taxa in the ICTV Master Species List (MSL) #38 prior to 2024 ratification

^b^Total number of taxa after the 2024 ratification vote (listed in the ICTV MSL #39)

## Principal changes to virus taxonomy

The 2024 round of taxonomy proposals saw the near completion of the mandated change of species names to a binomial format (i.e., genus + species epithet) [204–206]. This accounted for the vast majority of the 2884 species name re-assignments (Table 1).

There were no major reorganisations of or additions to the higher ranks of ICTV taxonomy, with retention of the six originally proposed realms and 10 kingdoms (Table 1). There was, however, the important addition of a new phylum, *Ambiviricota*, to which a large number of mobile genetic elements infecting fungi, previously introduced into the literature as “ambiviruses”, were assigned [209]. These unusual viruses with circular RNA genomes of approximately 5 kb combine features of both typical RNA viruses and viroids. On the one hand, they encode a canonical, albeit highly divergent, RNA-directed RNA polymerase that is homologous to those of other RNA viruses in the realm *Riboviria* [207, 210]. On the other hand, their genomes also possess ribozymes in various combinations in both the sense and antisense orientation, consistent with a rolling-circle mechanism for genome replication found in deltaviruses (family *Kolmioviridae* [208] classified in the realm *Ribozyviria*) and viroids. The proposal [160] assigns ambiviricots to four different families and 20 species.

At lower ranks, taxonomy proposals considered by subcommittees for animal DNA viruses and retroviruses, animal viruses with dsRNA and -ssRNA genomes, bacterial viruses, and fungal and protist viruses entailed a considerable expansion in the number of virus orders, families, genera, and species within a range of virus groups. This notably included viruses assigned to the *Cressdnaviricota*, a phylum of eukaryotic small DNA viruses (realm *Monodnaviria*), which nearly doubled in terms of the number of included virus families (from 12 to 23); negative-stranded RNA viruses (phylum *Negarnaviricota*); and bacterial and archaeal viruses in the class *Caudoviricetes* that were previously classified into families based on tail morphology and are now being placed into a much larger number of families and orders based on metrics of genome relatedness [211]. So far, this process has led to the establishment of 74 new families and seven orders. In addition, *Ghabrivirales*, an order for dsRNA viruses mainly infecting fungi, underwent a major revision [168].

A general proposal [202] described the organisational changes required to coordinate ICTV meeting and election cycles with those of the International Congresses of Virology (ICV) organized by the International Union of Microbiological Societies (IUMS). Elections to the ICTV EC had previously coincided with the ICV meetings, but the change to a two-year cycle of ICV meetings conflicts with the established three-year plenary sessions and election of the ICTV. Following the ratification of this general proposal, the ICTV will retain its three-year cycle; in the years when it coincides with the ICV meetings, the ICTV plenary meeting will be held in conjunction with the ICV. Otherwise, the plenary meeting will be held online after the EC annual meeting.

## Implementation and access

The latest set of proposals approved by the EC was made available on the ICTV website in April 2024 as a single zip file and in a directory of individual files at https://ictv.global/files/proposals/approved, indexed by virus group and subcommittee.

Updated versions of the Master Species List (up to version 39), which list all of the currently approved taxa (Table 1), can be accessed on the ICTV website at https://ictv.global/msl**.** A similarly updated version 39 of the Virus Metadata Resource (VMR) is located at https://ictv.global/vmr. This resource provides details of exemplar virus isolates for each species including GenBank accession numbers.

## Data Availability

All Taxonomy Proposals and all other ICTV resources mentioned in this article are freely available at the ICTV website (https://ictv.global/).
